# The dynamic transcriptome and metabolomics profiling in *Verticillium dahliae* inoculated *Arabidopsis thaliana*

**DOI:** 10.1038/s41598-018-33743-x

**Published:** 2018-10-18

**Authors:** Xiaofeng Su, Guoqing Lu, Huiming Guo, Kaixuan Zhang, Xiaokang Li, Hongmei Cheng

**Affiliations:** Biotechnology Research Institute, Chinese Academy of Agricultural Sciences, Beijing, 100081 China

## Abstract

*Verticillium* wilt caused by the soil-borne fungus *Verticillium dahliae* is a common, devastating plant vascular disease notorious for causing economic losses. Despite considerable research on plant resistance genes, there has been little progress in modeling the effects of this fungus owing to its complicated pathogenesis. Here, we analyzed the transcriptional and metabolic responses of *Arabidopsis thaliana* to *V. dahliae* inoculation by Illumina-based RNA sequencing (RNA-seq) and nuclear magnetic resonance (NMR) spectroscopy. We identified 13,916 differentially expressed genes (DEGs) in infected compared with mock-treated plants. Gene ontology analysis yielded 11,055 annotated DEGs, including 2,308 for response to stress and 2,234 for response to abiotic or biotic stimulus. Pathway classification revealed involvement of the metabolic, biosynthesis of secondary metabolites, plant–pathogen interaction, and plant hormone signal transduction pathways. In addition, 401 transcription factors, mainly in the MYB, bHLH, AP2-EREBP, NAC, and WRKY families, were up- or downregulated. NMR analysis found decreased tyrosine, asparagine, glutamate, glutamine, and arginine and increased alanine and threonine levels following inoculation, along with a significant increase in the glucosinolate sinigrin and a decrease in the flavonoid quercetin glycoside. Our data reveal corresponding changes in the global transcriptomic and metabolic profiles that provide insights into the complex gene-regulatory networks mediating the plant’s response to *V. dahliae* infection.

## Introduction

The soil-borne fungus *Verticillium dahliae* is responsible for widespread and devastating vascular disease in more than 200 species of dicotyledonous plants^1^. *V. dahliae* attacks susceptible plants through the roots, colonizes the plant vascular (xylem) system, and causes the death of aerial tissues^2^. The most typical symptom of *Verticillium* disease, generally referred to as *Verticillium* wilt, causes tremendous yield losses in many economically important crops^3^. *Verticillium* wilt is difficult to combat owing to the long-term survival of *V. dahliae* in the soil and the lack of fungicides with which to treat infected plants^2^. Currently, the preferred strategy to combat *Verticillium* wilt is the use of genetically improved *Verticillium*-resistant cultivars.

Plant resistance relies on the recognition of specific pathogen effector molecules by host plant resistance (R) proteins^4^. The first genetic locus found to be responsible for resistance against race 1 strains of *V. dahliae*, referred to as the *Ve* locus, was cloned in tomato (*Solanum lycopersicum*) and encodes cell surface receptor proteins^5^. The locus contains two closely linked and inversely oriented genes, *Ve1* and *Ve2*, of which only *Ve1* provides *V. dahliae* resistance in tomato^6^. The identification and functional characterization of *Ve* homologues was later extended to other plant species to include *SlVe1* from *S. lycopersicoides*^7^, *StVe* from *S. torvum* Swartz^8^, *mVe1* from *Mentha longifolia*^9^, *GbVe* from *Gossypium barbadense*^10^, *VvVe* from *Vitis vinifera*^11^, and *NgVe1* from *Nicotiana glutinosa*^12^. Virus-induced gene silencing in tomato revealed that *EDS1*, *NDR1*, *MEK2*, and *SERK3*/*BAK1* all act downstream of *Ve1* and are required for resistance to *V. dahliae*^6^. The requirement for *AtEDS1*, *AtNDR1*, and *AtSERK3*/*BAK1* for *Verticillium* resistance in *Arabidopsis thaliana* revealed that the critical signaling components used by *Ve1* are conserved^13^.

Silencing of *GhNDR1*, *GhMKK2*, and *GbEDS1* in cotton (*Gossypium hirsutum*) results in greater susceptibility to *V. dahliae*, suggesting that similar signaling cascades of Ve-mediated resistance exist in various species^14,15^. Gain- and loss-of-function mutations affecting a DNA-binding protein, AHL19, resulted in positive regulation of *Verticillium* wilt resistance in *Arabidopsis*^16^. A novel cotton subtilase, GbSBT1, recognizes a prohibitin-like protein secreted from *V. dahliae* and regulates *Verticillium* wilt resistance^17^. Further studies have been conducted on the role of transcription factors in *Verticillium* resistance. An ethylene-responsive GbERF1-like transcription factor contributes to resistance to *V. dahliae* in cotton by activating the expression of lignin biosynthesis genes^18^. GhATAF1, a NAC transcription factor, and GhMYB108 were both induced by *V. dahliae* infection and promote defense responses^19,20^.

Although various resistance genes have been functionally identified in the *Verticillium* resistance system, little is known about the complex molecular mechanisms underlying defense responses. Next-generation sequencing technologies offer fascinating opportunities to better understand the molecular networks of plant–pathogen interactions^21^. High-throughput RNA sequencing (RNA-seq), which does not require prior knowledge of genome sequences, has been used to obtain transcriptome changes in response to *V. dahliae* infection. RNA-seq analysis revealed 3,442 defense-responsive genes from the transcriptomic profiles of *V. dahliae*-infected cotton^22^. Further investigation of the expression of these genes revealed a critical role of lignin metabolism in the resistance of cotton to *Verticillium* wilt^23^. A comparison of RNA-seq results from infected sea-island and upland cotton to those from uninfected cotton revealed 44 differentially expressed genes (DEGs)^24^. A full-length cDNA library construction and expressed sequence tag (EST) sequencing in cotton challenged with *V. dahliae* identified 3,027 defense-related genes that are homologous to those in other plants, as well as 4,936 putative transcription factors^25^. Deep RNA sequencing of *V. dahliae*-infected *N. benthamiana* was performed to provide a catalog of transcripts produced by a *Solanaceous* model plant in response to pathogen attack^26^.

The use of a model plant-pathogen system could accelerate the discovery and understanding of the molecular mechanisms underlying *Verticillium* resistance. *Arabidopsis* possesses the first released genome sequence and the largest mutant collections. The conserved central components of the resistance signaling cascade have been reported, demonstrating that *Arabidopsis* is a suitable model to unravel the genetics of *Verticillium* resistance^27–29^. Therefore, the aim of this study was to use *Arabidopsis* as a model to identify transcriptome changes occurring during the process of *V. dahliae* infection. We examined *Arabidopsis* plants that had been infected with a highly toxic strain of *V. dahliae*, V991, at different time points after inoculation. We then performed transcriptomic analysis by RNA-seq and metabolomics analysis via NMR. We combined these data to analyze the expression of genes involved in signaling and metabolic pathways that are affected by *V. dahliae* inoculation.

## Results

### Establishment of experimental system

**Figure 1:**
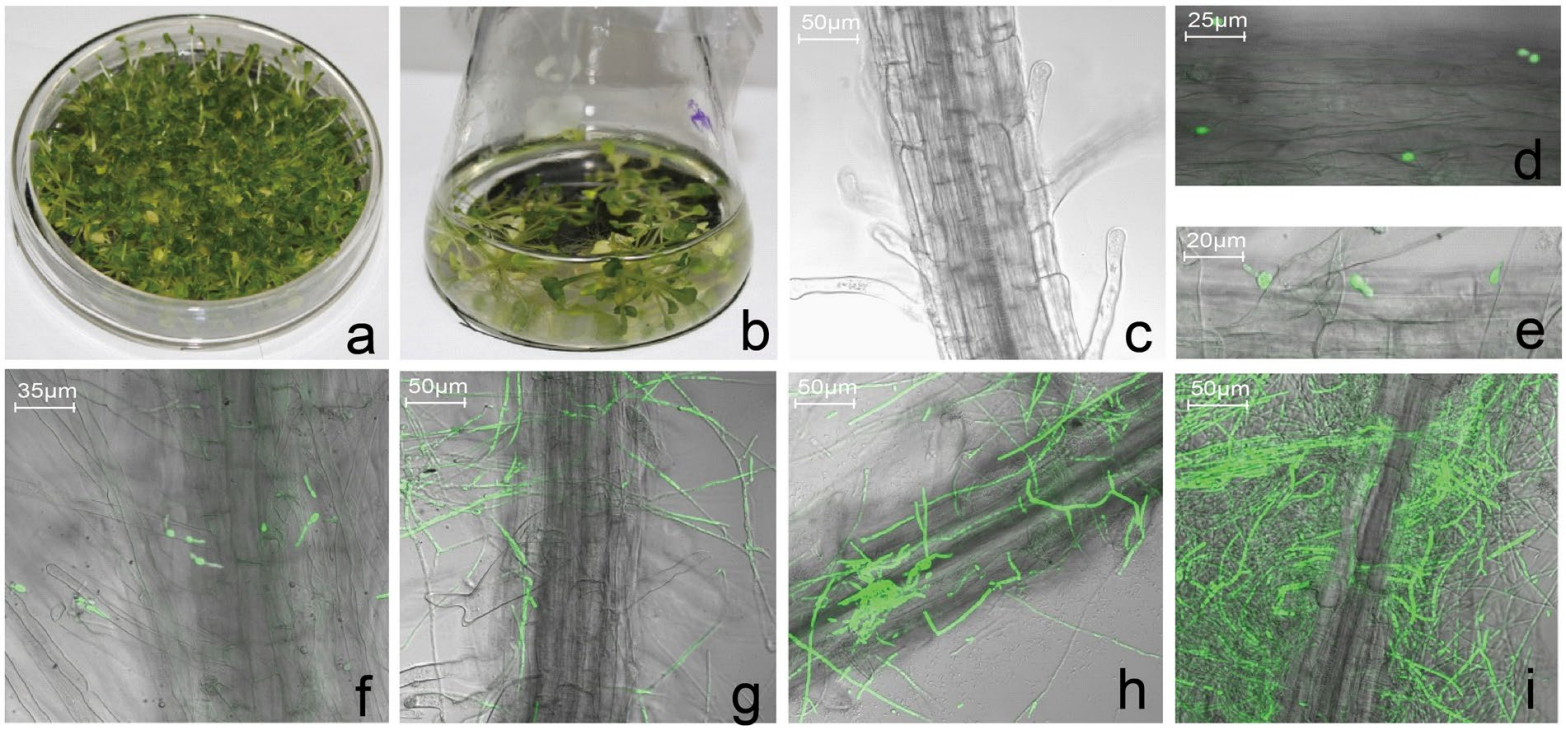
Colonization and infection of Vd-GFP on *Arabidopsis* roots. (**a**) The *Arabidopsis* seedlings were sown and grown on sterile MS agar medium. (**b**) Four-to-six-true-leaf seedlings were inoculated with 10^6^ spores/mL and transplanted to MS medium. Confocal micrographs of the roots were taken at (**c**) 0 hpi, (**d**) 4 hpi, (**e**) 8 hpi, (**f**) 12 hpi, (**g**) 24 hpi, (**h**) 48 hpi, and (**i**) 56 hpi.

To minimize the impacts of any other fungus and bacteria, we sowed the *Arabidopsis* seeds on MS agar medium (Fig. 1a). We then inoculated four-to-six-true-leaf seedlings with Vd-GFP spore suspension and transferred the plants into MS medium as described in Materials and Methods (Fig. 1b). To avoid *V. dahliae* overgrowth, the MS medium was changed every 4 h. The environmental impact of the experimental system on the plants was minimal and mock-inoculated plants were included as a control.

We observed the root surface of inoculated seedlings using a confocal microscope (Zeiss LSM 700, Jena, Germany) and found that the Vd-GFP spores were not attached to this surface (Fig. 1c). At 4 hour post inoculation (hpi), the conidia had colonized the root surface at random positions (Fig. 1d). At 8 hpi, a small quantity of spores had begun to germinate, with the germ tube forming from the merge of the conidium (Fig. 1e). At 12 hpi, many spores had germinated and the germ tube continued to elongate (Fig. 1f). At 24 hpi, germ tubes had developed into the longer mycelium, which extended over the surface of the root (Fig. 1g). At 48 and 56 hpi, the root was enclosed and entwined by a massive mycelium (Fig. 1h, i).

### *V. dahliae* infection affects metabolism

**Figure 2:**
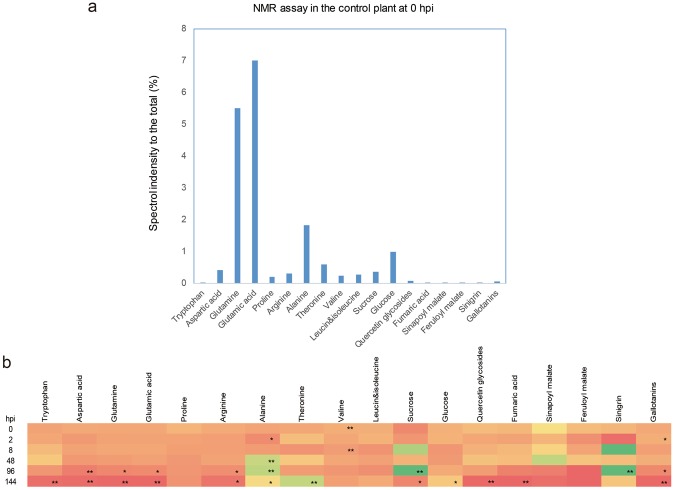
NMR analysis of inoculated plantlet from 0 hpi to 144 hpi. (**a**) Relative amounts of different compounds in the mock-treated sample at 0 hpi. (**b**) Dynamic changes in abundance of the compounds assayed over time. Color indicates the ratio of compound abundance in the inoculated samples to that in the mock-treated samples: red, 0; yellow, 1; and green, the maximum value (64.5, in sinigrin at 96 hpi). Single asterisk means *p* < 0.05; double asterisk means *p* < 0.01.

To determine how *V. dahliae* affects metabolism, we performed NMR analysis using plant materials harvested at 0, 2, 8, 48, 96, and 144 hpi with *V. dahliae* to assess metabolic changes. We assayed 19 substrates, including 11 amino acids, sucrose, glucose, quercetin glycoside, fumaric acid, sinapoyl malate, feruloyl malate, sinigrin, and gallotanins. At the beginning, glutamic acid was the most abundant, while glutamine was second; aspartic acid, proline, arginine, alanine, threonine, valine, sucrose, and glucose were present at moderate levels and the rest at low levels (Fig. 2a).

The NMR dynamics are demonstrated in Fig. 2b. After infection, the levels of tryptophan, aspartic acid, glutamine and glutamic acid, and arginine decreased over time. At 144 hpi, the tryptophan and aspartic acid levels in inoculated plants were around 10-fold lower than those in the mock-treated plants, arginine was 4-fold lower, and glutamine and glutamic acid were more than 20-fold lower. On the other hand, alanine and threonine levels increased as a result of *V. dahliae* infection, whereas proline, valine, leucine, and isoleucine did not show any marked variation.

The sucrose in the inoculated plantlets followed a more complex pattern, increasing from 0 to 96 hpi to a level around 5-fold higher than that in the mock-treated plants, but then decreasing until it dropped below that in mock-treated plants by 144 hpi. Furthermore, the glucose level in infected *Arabidopsis* did not show a substantial change in the period following inoculation with *V. dahliae*.

Along with the *V. dahliae* infection, the levels of quercetin glycoside, fumaric acid, and gallotanins in the plantlets decreased beginning at 96 hpi and were significantly lower than those in mock-treated plants at 144 hpi. Fumaric acid in the inoculated plantlet became undetectable at 144 hpi. Although the levels of sinapoyl malate and feruloyl malate also decreased, the decrease was not significant in comparison with the mock-treated plants. In addition, the level of sinigrin increased significantly, to almost 64-fold higher than that in the mock-treated plants (*P* < 0.01).

### Data analysis by Illumina sequencing platform

To obtain a detailed transcriptomic profile in the early infection stage, we extracted RNA from the mock-treated and *V. dahliae*-inoculated seedlings at 0, 0.25, 0.5, 1, 2, 4, 8, 12, and 24 hpi. We obtained a total of 505,069,964 clean reads from ten samples using the Illumina sequencing platform after adaptors and any low-quality sequences were removed. Alignment with the Arabidopsis database (www.arabidopsis.org) showed that the mapping rates of these reads were up to 82% in genomic sequences and 90% in expressed genes. Through assembly into contigs and unigenes and alignment, 33,096 expressed genes were generated in total. The data are shown in Table 2.

**Table 1:** Primer sequences used in the qRT-PCR experiments.

Gene	AGI	Sequences (5′-3′)
*PDF1.2*	AT5G44420	CTTGTTCTCTTTGCTGCTTTCG
		CATGTTTGGCTCCTTCAAG
*PR-1*	AT2G14610	TTCTTCCCTCGAAAGCTCAA
		AAGGCCCACCAGAGTGTATG
*VSP2*	At5g24770	GATACGGAACAGAGAAGACC
		AGCTTCGAGATTGTCGAGAG
*EF-1a*	At5g60390	TGAGCACGCTCTTCTTGCTTTC
		GGTGGTGGCATCCATCTTGTTA
	AT1G65960	GGCGGTGGGAGTAGGGACAGTTGGT
		GGCTCCGGTGACAATGTTGGGTTTG
	AT3G12120	AGCGACTACCAATGGCTGGATGACA
		ATGCGTCCAAGAGGGTTGTTGAGGT
	AT4G24460	AGCTCACCACTTTCGGCTACGTCTT
		TCCATTTGGGTACATCCATCATTTC
	AT5G46490	ACTACCATTGCACGAGCTTTGTTTA
		ATCTATCTTTATGTTCGGCATCCTT
	AT1G43640	CATACGAGCTGAACTTGCTTGGAAC
		GTGAAATCTCCCGAGTGGTTGACTA
	AT2G45220	AAGCACGGCTCTAACCAATCTTGAC
		ACCAGGTTTGACCCACGAAGGGAAC
	AT3G10720	AAGATTCTTGGTGGTGGGAACTCAA
		ACGGACCGACGATCACTGCTTTACT
	AT3G55770	ACTTTAATTTGCATGTAACAGTCGA
		TAAGTGGTTGTAACTTCCCTTCTCC

AGI, Arabidopsis Genome Initiative.

**Table 2:** Number of Arabidopsis reads used for the digital expression analysis.

Sample name	Clean reads	Genome map rate	Gene map rate	Expressed genes
Mock	50,449,400	84.26%	93.81%	28,125
0 hpi	50,494,868	84.27%	92.58%	28,673
0.25 hpi	50,496,898	83.36%	93.22%	28,143
0.5 hpi	50,566,258	82.63%	93.30%	27,711
1 hpi	50,524,584	82.42%	90.15%	28,277
2 hpi	50,536,484	82.19%	93.42%	28,090
4 hpi	50,516,548	83.33%	93.37%	27,989
8 hpi	50,511,294	84.68%	92.14%	27,753
12 hpi	50,565,214	84.01%	93.30%	27,942
24 hpi	50,408,416	83.82%	92.77%	27,911
Total number	505,069,964			280,614

### Differentially expressed gene (DEG) analysis in *A. thaliana*

To characterize *Arabidopsis* transcriptional responses to *V. dahliae* infection, we identified the unigenes whose expression levels changed significantly upon inoculation. The expression level was calculated in fragments per kilobase of transcript per million mapped reads (FPKM). To achieve this, we compared all the inoculated samples with the mock-treated samples. We found that transcription levels varied throughout the inoculation process. In response to the fungal stimulus and inoculation, a total of 13,916 genes showed differential expression (DEG) for at least one of the eight time points after inoculation. Some were identified as DEGs at more than one time points, and a subset demonstrated a different pattern at more than two time points. For example, the gene AT1G01100.1 was downregulated at 0.25 hpi, but upregulated at 1 hpi.

**Figure 3:**
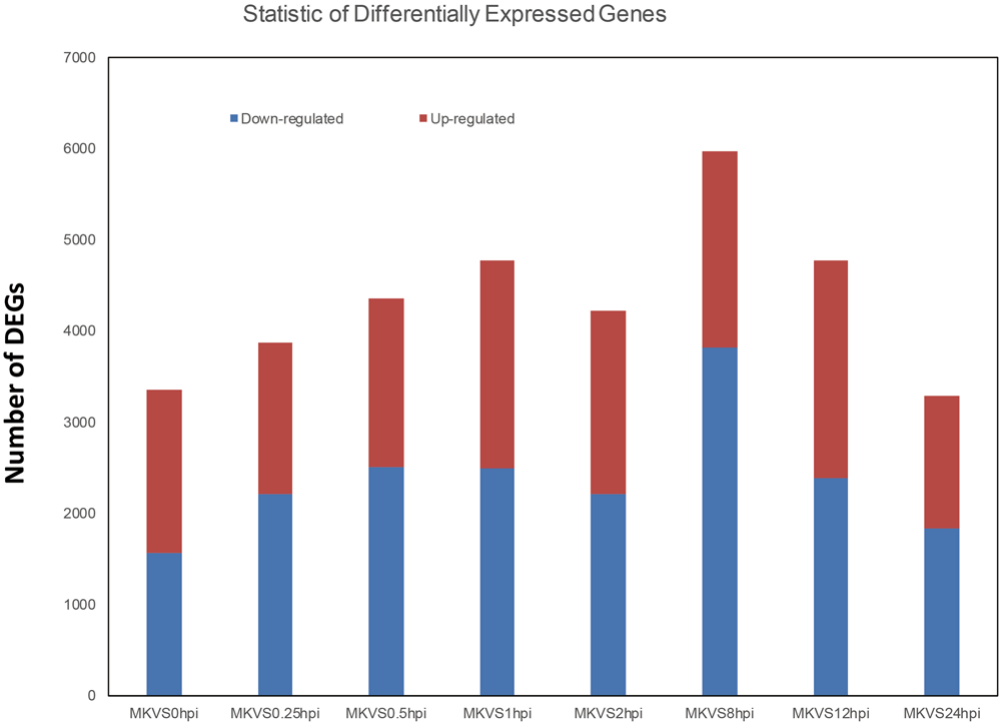
Numbers of DEGs down- and upregulated in the process of *V. dahliae* infection. MK, mock treatment; VS, versus; hpi, hour(s) post inoculation.

We separated the DEGs into downregulated and upregulated groups (Fig. 3). The highest level of DEG (>6,000) was achieved at 8hpi, of which the majority were downregulated. The smallest number of downregulated genes was found at 0 hpi, but that time point still had more than 3,000 DEGs.

All of the DEG data are presented in Additional File 1.

### Gene ontology (GO) enrichment analysis of DEGs

**Figure 4:**
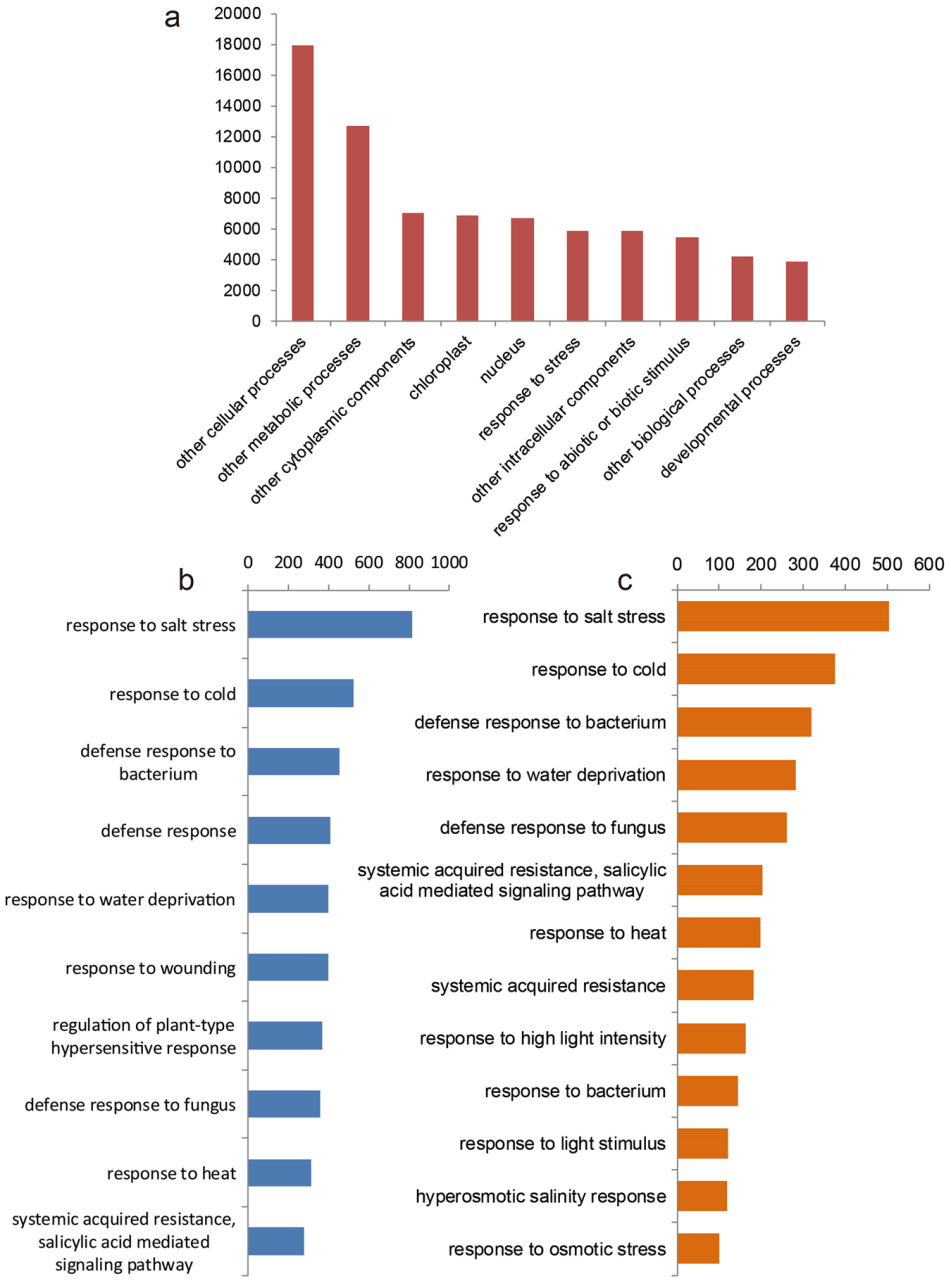
GO functional classification of the DEGs identified in this study. (**a**) Distribution of the DEGs among GOslim terms. (**b**) GO functional classification within the GOslim term ‘response to stress’. (**c**) GO functional classification within the GOslim term ‘response to abiotic or biotic stimulus’.

GO analysis was carried out to classify the functions of the differentially expressed genes. Based on Blast and SwissProt results, 11,055 DEGs were further annotated. All these annotated genes could be grouped into 46 categories according to their GOslim term. Among the top 10 categories, ‘response to stress’ containing 2,308 DEGs and ‘response to abiotic or biotic stimulus’ containing 2,234 DEGs ranked eighth and tenth, respectively (Fig. 4). Among the DEGs classified in ‘response to stress’, GO term identification showed that the most abundant was ‘response to salt stress’, with ‘respond to cold’ ranked second, ‘defense responds’ fourth, ‘defense response to fungus’ eighth, and ‘salicylic acid mediated signaling pathway’ tenth (Fig. 4b). Among the DEGs classified in ‘response to abiotic or biotic stimulus’, the ranking was quite similar, with ‘response to salt stress’ again ranked first, but ‘defense response to fungus’ ranked fifth and ‘salicylic acid mediated singling pathway’ sixth (Fig. 4c). All of the GO data are presented in Additional File 2.

### Pathway classification of DEGs

**Figure 5:**
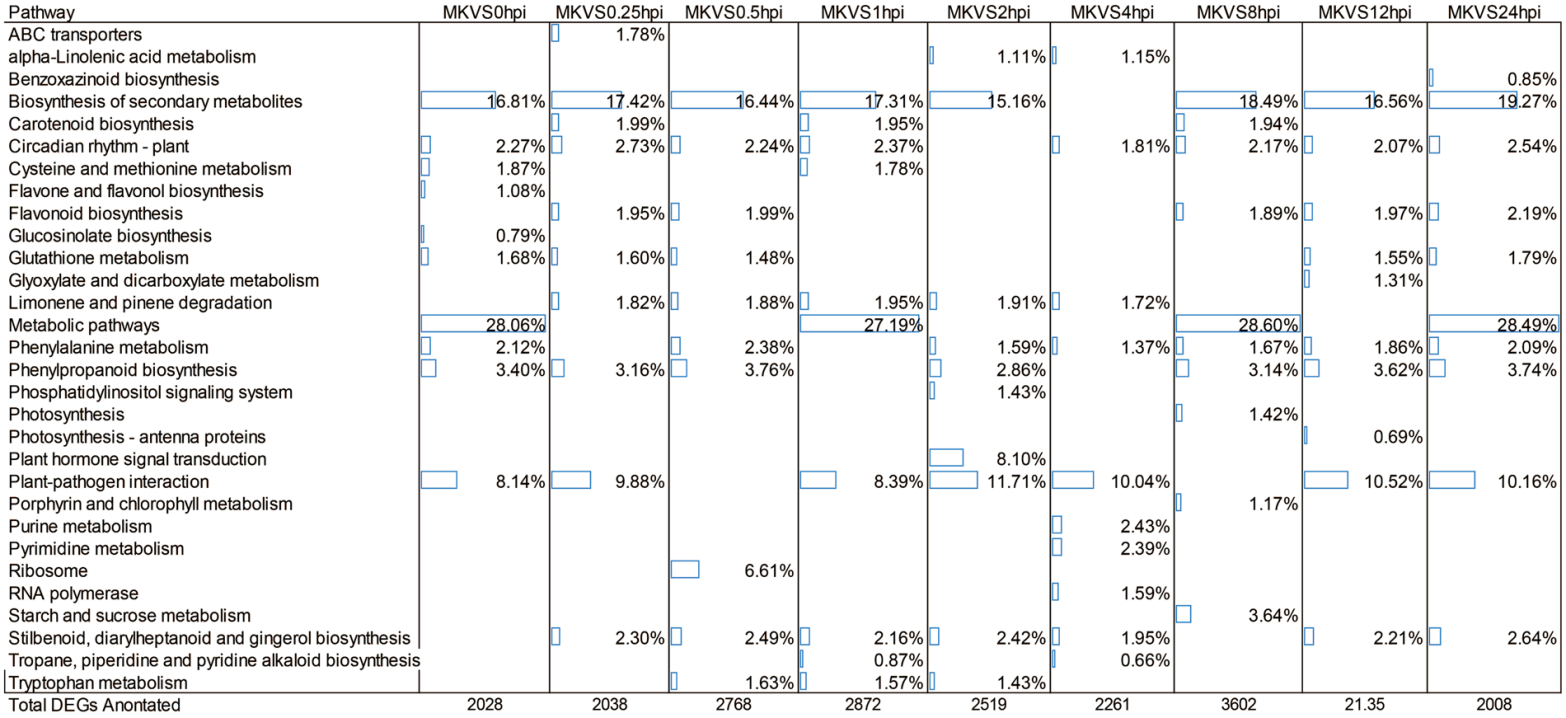
Statistical analysis of pathway enrichment at all sampling time points. The top 10 most enriched pathways are shown. The percentage and the corresponding bar refer to the ratio of the number of detected genes to the total number of genes annotated in this pathway. MK, mock treatment; VS, versus; hpi, hour(s) post inoculation.

DEGs and all the genes assembled were analyzed with pathway tools in the KEGG database. From 0 hpi to 24 hpi, there were 2,008–3,636 DEGs annotated to be involved in 55 different biochemical pathways, as showed in Additional File 3. The top 10 enriched pathways at all nine sampling time points are shown in Fig. 5, while the top 20 were showed in Additional File 3. The biosynthesis of secondary metabolism was the category with the greatest enrichment, and metabolic pathway and plant–pathogen interaction were also very highly enriched. Plant hormone signal transduction, ribosome, starch and sucrose metabolism, tryptophan metabolism, circadian rhythm, glutathione metabolism, phenylalanine metabolism, carotenoid biosynthesis, and flavonoid biosynthesis also showed marked enrichment.

Among these pathways, those of amino acid metabolism were found. For example as mentioned above, the tryptophan metabolism pathway were highly enriched (included in the Top-20) in every time point checked, which coincided with the NMR data somehow. The enrichment in alanine, aspartate and glutamate metabolism pathway was also remarkable in some time points; on the other hand phenylalanine metabolism, and phenylalanine, tyrosine and trypophan biosynthesis were highly enrich as well.

### Identification of putative transcription factors

**Figure 6:**
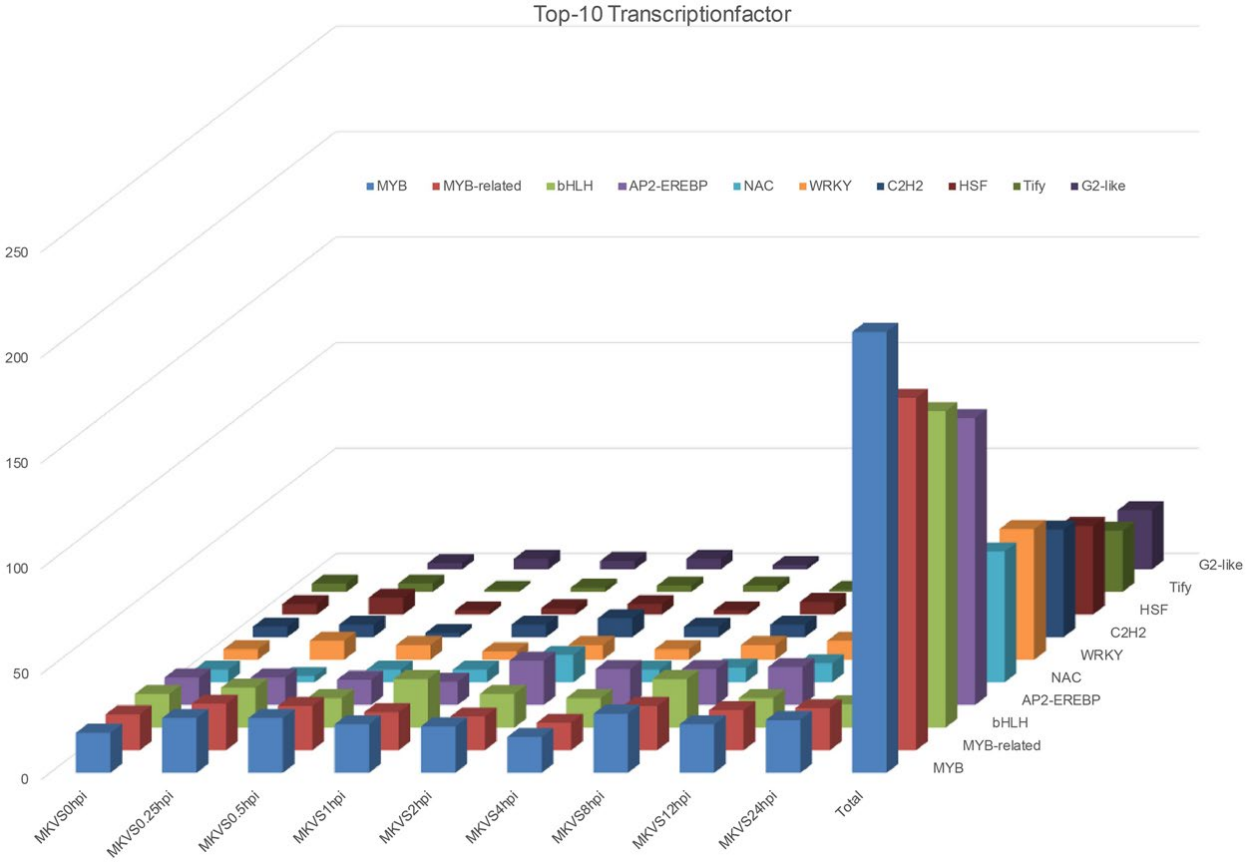
DEGs predicted to encode transcription factors. Among the DEGs from 0 hpi to 24 hpi, 477 independent genes were identified as putative transcription factors (TFs). The top 10 putative TFs identified are shown in the chart. MK, mock treatment; VS, versus; hpi, hour(s) post inoculation.

Transcription factors mediate the expression levels of a series of genes and are tightly associated with pathways. Therefore, they can contribute both to the biological processes of normal development and to resistance against biotic and abiotic stimuli in plants^30,31^. Among the DEGs from 0 hpi to 24 hpi, we identified 477 independent genes as putative transcription factors by searching PlantTFDB2.0 from a comprehensive plant transcription factor database (PlantTFDB; http://planttfdb.cbi.pku.edu.cn). The most abundant family of transcription factors was MYB (15.72%), followed by MYB-related (12.79%), bHLH (10.48%), AP2-EREBP (10.06%), NAC (5.24%), WRKY (4.61%), C2H2 (3.56%), G2-like (2.31%), MADS (2.31%), and HSF (2.10%) (Fig. 6). All of the data for the transcription factors can be found in Additional File 4.

### *V. dahliae*-induced plant defense gene expression

**Figure 7:**
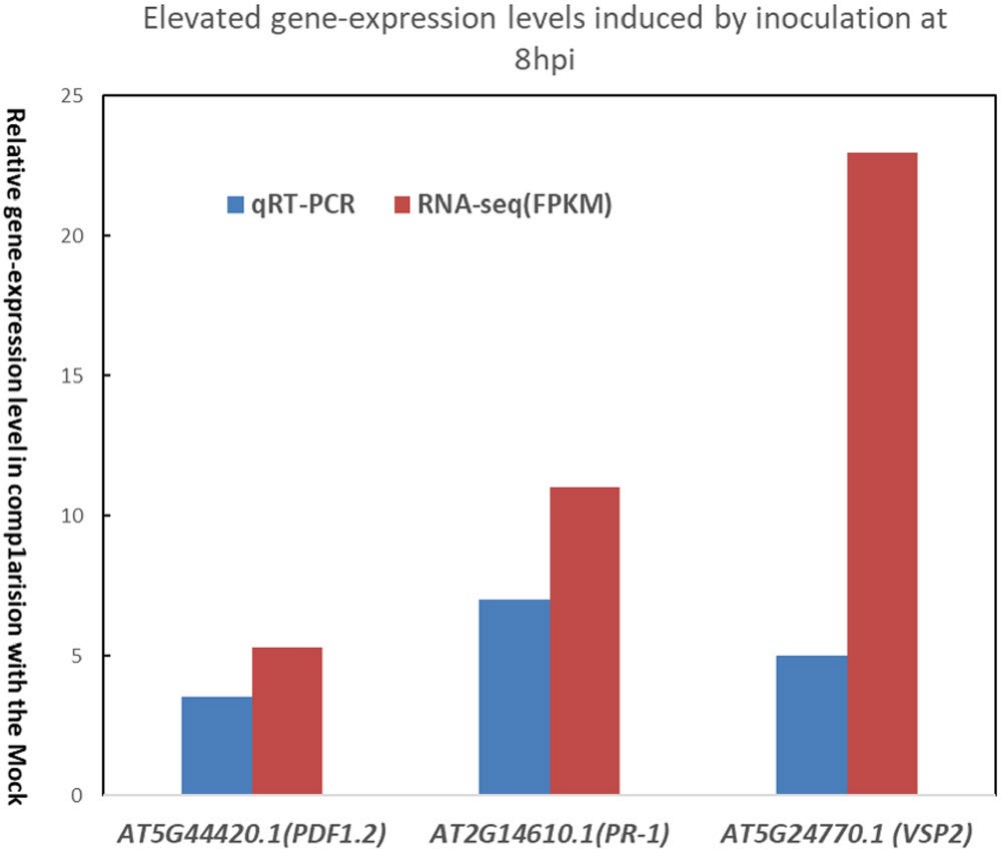
Expression levels of special genes in different treated groups. The salicylic acid (SA), jasmonic acid (JA), and ethylene (ET) signaling pathways mediate innate immune responses. The expression of the marker genes *AT5G44420.1*, *T2G14610.1*, and *AT5G24770.1* in response to *V. dahliae* infection was monitored by qRT-PCR at 8 hpi time-point. *AT5G44420.1* coding plant defensing (PDF1.2); *AT2G14610.1* coding pathogenesis-related gene (PR-1); *AT5G24770.1* coding vegetative storage protein 2 (VSP2).

Given that the salicylic acid (SA), jasmonic acid (JA), and ethylene (ET) signaling pathways mediate innate immune responses, we decided to investigate the expression of marker genes of these pathways in response to *V. dahliae* infection according to the RNA seq data as well as qRT-PCR. We picked the time point of 8 hpi, and *PR-1* (*AT2G14610.1*), *VSP2* (*AT5G24770.1*), and *PDF1.2* (*AT5G44420.1*) as the marker genes for SA, JA and ET. All these three genes showed expression level elevation, validated by both methods, when the incretion assayed by qRT-PCR were generally higher than that of RNA-seq (Fig. 7). Later we chose four genes of up-regulated and another four of down regulated identified by RNA-seq, for further validation of qRT-PCR. The qRT-PCR results were in accordance with the RNA-seq result. The expression levels of these genes and their description were showed in Table 3.

**Table 3:** The gene-expression level variation induced by inoculation.

Gene ID	Time	Inoculation induced Variation	RNA-Seq	qRT-PCR
AT1G65960	12 hpi	Down	0.77	0.59
AT3G12120	1 hpi	Down	0.97	0.43
AT4G24460	8 hpi	Down	0.55	0.92
AT5G46490	2 hpi	Down	0.26	0.37
AT1G43640	8 hpi	Up	20.00	6.29
AT2G45220	12 hpi	Up	16.40	9.21
AT3G10720	1 hpi	Up	1.30	8.43
AT3G55770	12 hpi	Up	1.46	2.15

Note: RNA-Seq means ratio between FPKM values of treatment and the mock, qRT-PCR means ratio between relative expression level of treatment and the mock assayed in qRT-PCR test; *AT1G65960.2* coding glutamate decarboxylase 2; *AT3G12120.1* coding fatty acid desaturase 2; AT4G24460.1 coding CRT (chloroquine-resistance transporter)-like transporter 2; *AT5G46490.2* coding Disease resistance protein (TIR-NBS-LRR class) family; *AT1G43640.1* coding tubby like protein 5; *AT2G45220.1* coding pectin methylesterase 17; *AT3G10720.1* coding plant invertase/pectin methylesterase inhibitor superfamily; *AT3G55770.7* coding *Arabidopsis* LIM protein.

## Discussion

*Verticillium* wilt causes significant economic losses worldwide owing to the lack of effective fungicides and the limited knowledge of *Verticillium* resistance mechanisms. The susceptibility of *Arabidopsis* to *V. dahliae* infection makes it possible to use this species as a model to provide increased insight into plant-pathogen recognition and the complex signaling networks involved in defense responses^2,28^. The high-throughput deep RNA sequencing (RNA-seq) of *V. dahliae*-infected *Arabidopsis* at different time points provided multiple insights into the genetic basis of *Verticillium* resistance. Interactions between the plant host and pathogen were observed through the whole infection. Even in the very early stage of infection, DEGs were detected. In 0 hpi, 0.25 hpi, 0.5 hpi and 1 hpi, the amount of DEGs kept increasing. It is remarkable because that fungal spores germinate and penetrate the epidermal cells of *Arabidopsis* roots within the first 12 hpi (Fig. 1d–f), which is visible and in accordance with previous data^2,23^. However, the DEGs data showed that the plant gave response in quite earlier stage. Even in 0 hpi when the plants were just inoculated in spore suspend after 2 min, there were more than 3000 DEGs taken into account. These DEGs might be caused by the staining (as descript in Materials and Methods), and it was also possibly the quick respond from *Arabidopsis* plantlet to the exogenous pathogen.

The initial step in the plant response to pathogen challenge is recognition of the non-self pathogen signals by the plant R protein. Up to date, only from tomato has a cell surface receptor, Ve1, been well characterized functionally, and this is thought to mediate *Verticillium* resistance^5,6^. However, the *V. dahliae* Ave1 effector induces various defense genes independently of Ve1 in tomato, suggesting that the signal might be transduced by another receptor^32^. In the present research, the pathway classification of DEGs identified a total of 2,308 DEGs involved in the plant’s response to stress. Of these, 453 DEGs are associated with defense response, 369 with regulation of the plant-type hypersensitive response, and 358 with the defense response to fungi. The largest group of DEGs was that comprising genes associated with response to salt stress, with other large groups being associated with cold stress response and the defense response to bacteria. GO annotation showed that many of the DEGs we identified encoded proteins with overlapping functions in multiple biotic or abiotic stresses. This implies that although some DEGs were identified as related to response to stresses other than fungi (such as salt, water deprivation, and wounding), they may also have unknown functions relevant to *V. dahliae* infection and may thus provide information that could help uncover other Ave1 receptors.

After the Ave1 effector is detected by the plant cell, signal transduction is carried on by phytohormone signaling pathways. SA is known to be effective against biotrophic pathogens while JA and ET are effective against necrotrophs^33^. The SA and JA signaling pathways are required for a form of *Verticillium* resistance induced by the nonpathogenic bacterium *Paenibacillus alvei*^34^. The pattern of increased expression of *PR-1*, *VSP2*, and *PDF1.2* validated by RNA-seq as well as qRT-PCR confirmed the involvement of SA and JA signaling in defense responses to *V. dahliae* (Fig. 7). The RNA-seq data also showed ethylene biosynthetic gene *AOC1* (*AT4G35830*) were triggered by the inoculation, its expression level kept up-regulated in almost all of the time-points. Among the DEGs from the entire infection process, we identified 413 genes involved in the SA hormone signaling pathway and 404 genes involved in JA signaling. Among the 413 DEGs associated with SA signaling, 162 are involved with SA-mediated systematic acquired resistance; among the DEGs associated with JA signaling, 11 are associated with JA-signal-mediated induced systematic resistance; and among the ethylene associated genes, 13 DEG are involved in jasmonic acid and ethylene-dependent systemic resistance. The expression of defense-related genes is mediated by transcription factors modulating the network of stress responses. A total of 477 putative transcription factor coding genes were identified among the DEGs, among which MYB and MYB-related transcription factors were the most abundant (Table 3). These transcription-factor-encoding DEGs could serve as a reference for the further analysis of *V. dahliae* and thereby contribute to the understanding of the complex signaling network of the plant’s response to pathogen invasion.

The host plant’s response to pathogens involves a systematic network. The pathogen-associated expression patterns of pivotal genes are closely related to primary and secondary metabolism. In the defense response to *V. dahliae*, for example, carbohydrate and nitrogen metabolism are reprogrammed and photosynthesis processes are depressed^30^. Xu *et al*. reported with PCR-select suppression subtractive hybridization, it is proved that *V. dahliae* inoculation on cotton resulted in DEGs mainly involved in metabolism, stress⁄defence response, cell structure and signal transduction^22^, when present research found quite similar phenomenon. Mo *et al*. found that Spermine biosynthesis is required for the SA-mediated signaling involved in adaptation to *V. dahliae* colonization by screening suppression subtractive hybridization and cDNA libraries of cotton (*Gossypium*) species tolerant to Verticillium wilt^31^. To date, there had been little information about the metabolomics of *V. dahliae*-infected plants. We therefore expected that an investigation of the relationships between metabolomics and resistance could promote understanding of *Verticillium* wilt resistance. In our present research, we used NMR analysis to monitor metabolic changes following inoculation with *V. dahliae*. Previous research had indicated that biotic and abiotic stress influences the homeostasis of free amino acids; in *Arabidopsis*, inoculation with *P. syringae* bacteria resulted in decreases in the abundance of branched-chain amino acids (valine, leucine, and isoleucine), aromatic amino acids (phenylalanine, tyrosine, and tryptophan), and aspartate^35^. In present research we did find the trend of decreasing in aromatic amino acid tryptophan after inoculation but not in the branched-chain amino acid. The metabolism variation assayed by NMR in present research showed that tryptophan content of the plantlet showed a dramatically dropdown due to the *V. dahliae* infection. Simultaneously, the transcriptomic data also showed that the tryptophan metabolic pathway genes were highly enriched in all of the time points. The gene (*AT4G23100.2*) coding the key enzyme (γ‐glutamylcysteine synthetase) of glutathione biosynthesis and tryptophan metabolism was the DEG of up regulated in 0 hpi, 0.25 hpi, 0.5 hpi, 2 hpi, 8 hpi and 12 hpi. As reported previously, glutathione is very important for plant resistant to diseases^36,37^. Simultaneously, aspartic acid, glutamine and glutamic acid were reduced especially in the late stage, but alanine increased. All these amino acid are involved in alanine, aspartate and glutamate metabolism pathway which is remarkably enriched in transcriptomic analysis (Additional File 3). The gene coding (*ATG17290*) alanine catalysis key enzyme alanine amino transferase, were highly expressed in 8 hpi. The variation in alanine, aspartate and glutamate metabolism could be induced by the fungal growth, but also possible due to the anaerobic stress^38^ in submerge treatment (as described in materials and methods part). Another metabolic pathway that has been found, in cotton, to be very important for *Verticillium* wilt resistance is lignin metabolism^23^. Lignin biosynthesis begins in the cytosol with the synthesis of glycosylated monolignols from phenylalanine. The RNA-seq data showed that phenylalanine metabolism highly enriched. Phenylalanine ammonia-lyse is an important plant enzyme to form trans-cinnamic acid, a precyrsor of lignin^39^. The genes (*AT3G10340.1* and *AT2G37040.1*) encoding the enzyme showed elevated expression level in 8 hpi, 12 hpi and 24 hpi. We measured the levels of three precursors (fumaric acid, sinapoyl malate and Feruloyl malte) of lignin biosynthesis in *V. dahliae*-inoculated *Arabidopsis*. We found all of these three substances increased until 48 hpi and then kept decreasing difference. The transcriptome data showed the expression level of genes *AT5G54160.1* and *AT4G36220.1* were enhanced after inoculation (8 hpi, 12 hpi and 24 hpi as showed in Additional File 3). They encode caffeic acid/5-hydroxyferulic acid O-methyltransferase and ferulate 5-hydroxylase, which are both essential in lignin biosynthesis^40^. On the other hand, the level of the glucosinolate sinigrin increased and that of the flavonoid quercetin glycoside decreased, while flavonoid and glucosinolate biosynthesis were also markedly enriched in transcriptomic analysis.

## Conclusion

Our present research provides comprehensive information on the metabolomic and transcriptomic changes occurring in *Arabidopsis* inoculated with *V. dahliae*. The metabolomic profile of the infected plantlets differed significantly from that of the mock-infected plantlets; in addition, a large number of DEGs were identified based on transcriptomic analysis. The dynamic events that follow fungal inoculation highlighted many functional genes and the pathways in which they are involved. The copious data we obtained, and the correspondence between the metabolome and transcriptome they imply, should serve as a useful reference in efforts to decipher the complexity of the interactions between plants and this devastating pathogen.

## Materials and Methods

### Fungal strain and inoculum preparation

The highly virulent *V. dahliae* strain V991 was kindly provided by Dr. Guiliang Jian of the Institute of Plant Protection, Chinese Academy of Agricultural Sciences (CAAS), Beijing, China. The pathogen was cultured in liquid complete medium (CM) with 50 mg/L ampicillin and 50 mg/L kanamycin at 25 °C. After five days, the culture was filtered through 0.4 µm mesh and centrifuged at 10,000 rpm for 5 min, and the supernatant was discarded. The number of spores was counted with a light microscope (OLYMPUS BX52, Tokyo, Japan) and the extract was diluted to 10^6^ spores/mL to produce the spore suspension used for inoculation.

### Plant materials and inoculation

Sterile seeds of *Arabidopsis thaliana* (ecotype Columbia 0) were sown on Murashige-Skoog (MS) agar medium and cultured at a photoperiod of 16 h light (24 °C) and 8 h darkness (21 °C). Four-to-six-true-leaf seedlings were transferred into liquid MS medium. After 48 h, the seedlings were submerged in the prepared spore suspension for 2 min. Subsequently, the seedlings were laid on sterile filter paper to eliminate excess solution and transferred into fresh liquid MS medium.

To obtain a detailed transcriptomic profile in the early infection stage, we extracted RNA from the mock-treated and *V. dahliae*-inoculated seedlings at 0, 0.25, 0.5, 1, 2, 4, 8, 12, and 24 hpi. Meanwhile, the plant materials were prepared for metabolomics analysis at 0, 2, 8, 48, 96, and 144 hpi. To minimize systematic bias, the data were collected three times.

### cDNA preparation for RNA-seq

Twenty plants from each treatment were collected at 0, 0.25, 0.5, 1, 2, 4, 8, 12, and 24 hours post inoculation (hpi). Uninfected wild-type seedlings were designated the mock-treatment group. The samples were washed with sterile water and then quickly frozen in liquid nitrogen and stored at −80 °C.

Total RNA was isolated from the frozen tissues using an RNA Extraction Kit (YPHBio, Tianjin, China) following the manufacturer’s instructions, including an on-column DNase I digestion. The mRNA was further purified using oligo(dT) magnetic beads and a magnet separator. The quality and concentration of mRNA were determined using an Agilent 2100 Bioanalyzer (Agilent, California, America). Next, the mRNA was broken into small fragments by heat treatment, and the fragments were reverse transcribed into first-strand cDNA with random hexamer primers. The second-strand cDNA was synthesized and sequenced using a HiSeq^TM^ 2000 instrument (Illumina, America).

### Analysis of RNA-seq data

The raw sequences were processed to remove low-quality reads (with ratio of unknown sequences ‘N’ > 10%) and adaptor sequences using the LUCY programs^41^. Meanwhile, possible *Verticillium* sequences were removed from the raw data (www.broadinstitute.org). After the quality-control mock treatment had been carried out, the clean data were assembled into contigs and aligned with *Arabidopsis thaliana* data (www.arabidopsis.org). After clustering and assembly, similarities between ESTs (expressed sequence tags) and sequences were identified using the NCBI BLAST program, version 2.2.6^26^. Furthermore, the differentially expressed genes (DEGs) were subjected to gene ontology (GO) analysis^42^, pathway enrichment analysis^24^, transcription factor analysis, and cluster analysis^43^.

### Preparation of plant material for nuclear magnetic resonance (NMR)

The plant material collected at 0, 2, 8, 48, 96, and 144 hpi was ground and subsequently freeze-dried. The mock-treated samples were prepared at the same time points but without infection. Then, 20 mg of freeze-dried ground tissues was added to 1 mL of deuterium solvents, CH_3_OH-*d*_4_-KH_2_O_4_ buffer in D_2_O, which includes trimethylsilyl propionic acid sodium salt-*d*4 (0.01%, w/w, TMSP), and then extracted by ultrasonication for 10 min and centrifuged at 13,000 rpm (10,968 g) for 1 min. From the supernatant, 250 µL of mixture was transferred into 3-mm NMR tubes and measured in an NMR instrument (600 MHz Bruker DMX-600 spectrometer) at 25 °C.

### Analysis of NMR data

Deuterated methanol and water were purchased from Sigma-Aldrich (St. Louis, MO, USA). ^1^H NMR spectra were recorded at 25 °C on a 600 MHz Bruker DMX-600 spectrometer (Bruker, Karlsruhe, Germany) operating at a proton NMR frequency of 600.13 MHz for ^13^C. CD_3_OD was used as the internal control.

The ^1^H NMR spectra were automatically reduced to ASCII files. Spectral intensities were scaled to total or internal standards (TMSP or TMS signals at δ 0.0) and reduced to integrated regions of equal width (δ 0.04) corresponding to the region of δ 0.0–10.0. The regions of δ 4.7–4.9 and δ 3.28–3.34 were excluded from the analysis because of the residual signals of D_2_O and CD_3_OD, respectively. Bucketing was performed using AMIX software (ver. 3.0 Bruker) with scaling on total intensity. Projections to latent structures (PLS) and orthogonal PLS (OPLS) with scaling based on unit variance were performed with SIMCA-P + software (v. 13.3, Umetrics, Umeå, Sweden).

### Expression level analysis of candidate genes by qRT-PCR

Total RNA was isolated from the *Arabidopsis* plants using RNA extraction kits (YPHBio, Tianjin, China) according to the manufacturer’s instructions. The cDNA was synthesized using reverse transcription kits (TransGen, Beijing, China). qRT-PCR was carried out with a 7500 Real Time PCR System (Applied Biosystems, Foster City, CA, USA), using TransStart Top Green qPCR SuperMix (TransGen, Beijing, China) as described by the manufacturer. Primers used to identify the transcripts of specific genes are listed in Table 1. The relative expression levels were calculated using the 2^−∆∆Ct^ method^25^, with the *Arabidopsis* housekeeping gene (*EF-1a*, At5g60390) as the internal mock.

## Electronic supplementary material

Dataset 1

Dataset 2

Dataset 3

Dataset 4

## Data Availability

The NMR data and the RNA-seq information developed in present research are freely available to the research community. And the data generated or analysed during this study are included in Additional Files 1–4.
